# Ex-vivo Raman spectroscopy and AI-based classification of soft tissue sarcomas

**DOI:** 10.1371/journal.pone.0330618

**Published:** 2025-09-02

**Authors:** Maede Boroji, Vahid Danesh, David Barrera, Elizabeth Lee, Paul G. Arauz, Renee F. Farrell, Brendan F. Boyce, Fazel A. Khan, Imin Kao

**Affiliations:** 1 Department of Mechanical Engineering, Stony Brook University, Stony Brook, New York, United States of America; 2 Department of Pathology and Laboratory Medicine, Stony Brook University Hospital, Stony Brook, New York, United States of America; 3 Department of Orthopaedic Surgery, Stony Brook University Hospital, Stony Brook, New York, United States of America; 4 Department of Radiation Oncology, Stony Brook University Hospital, Stony Brook, New York, United States of America; IIIT Kurnool: Indian Institute of Information Technology Design and Manufacturing Kurnool, INDIA

## Abstract

Soft tissue sarcomas (STS) are a diverse and rare group of malignant tumors arising from the connective tissues of the body, including fibrous tissue, muscles, fat, nerves, and blood vessels. The heterogeneity and infrequency of these tumors pose significant challenges in both diagnosis and treatment. Surgical resection remains the primary treatment strategy, often complemented by radiation or chemotherapy, contingent upon the tumor’s size, location, and stage. However, current methods for assessing intraoperative margins are limited, underscoring the need for improved approaches that enhance both efficiency and accuracy. This study investigates the potential of microscopic Raman spectroscopy for distinguishing between different subtypes of soft tissue sarcomas, benign tumors, and normal tissue. Ex-vivo Raman measurements were conducted using a 633 nm excitation wavelength on samples obtained from surgical resections of seven patients (286,672 spectra). After pre-processing of the data, a custom ResNet architecture was developed to accurately classify the different tissue types, achieving an overall weighted accuracy of 97.1% and a clinical alert rate of 1.46%, a critical metric for quantifying the misclassification of malignant tissues. These findings suggest that single Raman spectra could serve as a rapid, non-invasive tool for surgical guidance, aiding in the precise identification of abnormal tissue types and margins.

## Introduction

Mesenchymal tumors are among the most challenging areas in diagnostic pathology. Refining classification schemes is crucial for enhancing the accuracy of pathological diagnoses, which in turn improves therapeutic options [1]. The spectrum of types of soft tissue tumors and related conditions is broad, encompassing benign, intermediate and malignant entities, each with unique histological characteristics. There are over 100 histologically different subtypes of soft tissue sarcomas (STS), highlighting the complexity and diversity of these tumors [2].

Among benign tumors, lipomas are the most common, consisting of mature adipose tissue. They can have variable histologic appearances, such as focal myxoid stroma, fat necrosis, or fibrosis, and may occur within muscles as intramuscular lipomas. In contrast, liposarcomas are malignant tumors arising from adipocyte precursors. These tumors are typically large and bulky, representing the most common subtype of STS, and accounting for 20% of all adult STS cases. Subtypes with greatest metastatic potential include dedifferentiated liposarcoma and pleomorphic liposarcoma. Metastatic dedifferentiated liposarcoma is commonly resistant to chemotherapy and radiation. Pleomorphic liposarcoma, the rarest subtype, comprises 5% of all liposarcomas and, like dedifferentiated liposarcoma, is aggressive with significant metastatic potential, contributing to increased disease-related mortality. Myxoid liposarcomas (another subtype of liposarcoma) constitute 30% of all liposarcomas and frequently occur in the lower extremities, with metastases observed commonly in the lungs, soft tissue, and bones [1,2].

While adipocytic tumors, such as lipomas and liposarcomas, primarily arise from adipose tissue, tumors arising from smooth muscle cells represent another significant category of soft tissue tumors. Leiomyomas are benign smooth muscle tumors, many of which occur in the uterus as fibroids. Leiomyosarcoma, a malignant counterpart, arises from smooth muscle cells in the uterus and other organs and in connective tissue, account for approximately 10% of all STS. The standard treatment for leiomyosarcoma is surgical resection when feasible, as early excision with wide margins offers the best chance for favorable outcomes. Despite this, leiomyosarcoma can remain undetected for extended periods, and the 5-year relapse rate after surgery is 40%, which is associated with a very high mortality rate [1,2].

Another subtype of STS is the fibroblastic/myofibroblastic tumors. As classified by the World Health Organization (WHO) [1], within this category, myxofibrosarcoma is a significant malignancy, primarily affecting the extremities of elderly patients and accounting for approximately 5% of all STSs. This type of sarcoma is typically characterized by a slowly enlarging, asymptomatic mass, which often complicates early detection and diagnosis, and it is characterized by frequent local recurrence [3,4].

According to the 2024 American Cancer Society report, approximately 13,590 new cases of STSs were diagnosed in the United States, with 57% of the cases occurring in males and 43% in females. Additionally, around 5,200 (38%) individuals are expected to die from soft tissue sarcomas in the United States [5].

Current treatments for STS typically involve a combination of modalities, such as surgery, radiation therapy, and chemotherapy. To effectively treat STSs, the primary approach is complete surgical resection of the tumor with a wide margin of normal tissue, aiming to leave no tumor cells behind. However, local recurrence remains a significant challenge if the tumor is not entirely removed with negative resection margins [6]. To mitigate this risk, it is crucial to have a reliable system for assessing the surgical margins after tumor resection. Currently, intraoperative frozen section histologic analysis is the standard method for margin assessment, but it is time-consuming, labor-intensive, and prone to sampling errors, as it only evaluates a small portion of the surgical bed. This process can take 30 to 60 minutes, during which the patient remains under anesthesia with an open wound [7,8]. While preoperative imaging techniques like magnetic resonance imaging (MRI) provide a general overview of the tumor’s size and location, they do not offer real-time margin evaluation during surgery. The ultimate margin status is determined postoperatively through comprehensive histopathologic examination, which can take several days to a week, potentially delaying further treatment if positive margins are found. Moreover, rarity of sarcomas can be a factor to negatively impact the accuracy and reproducibility of pathologic interpretation. With some types occurring at a rate as low as 0.1 cases per 100,000 individuals, pathologists who do not work in high-volume centers may encounter them very infrequently [1]. Therefore, there is an unmet clinical need for faster and more accurate methods to ensure complete tumor resections in real time, minimizing the risk of local recurrence and improving patient outcomes.

Research has been conducted on intraoperative Raman spectroscopy for a few subtypes of soft tissue sarcoma [7] and characterization of normal tissues such as white adipose tissues (WAT) in surgical sterile settings [9], utilizing a 785nm diode laser with a handheld probe. Previously, studies have also been published on the use of autofluorescence spectroscopy for in-vivo analysis of STS [10]. Other groups have investigated the use of Raman spectroscopy and coherent Raman scattering (CRS) microscopy in brain tumor surgery to improve excision accuracy by differentiating between normal and tumor-infiltrated tissues in real-time [11,12]. Additionally, Raman spectroscopy has been applied for disease diagnosis in various organs, including lung [13], skin [14,15], breast [16,17], kidney [18] and bladder [19].

Unlike many studies that broadly categorize all STSs as similar tumors or cancerous tissues [7,20], our approach recognizes the distinct histological characteristics of various STS subtypes, which is important from a pathological perspective. By treating each subtype as a separate category and developing a classification model to differentiate among them, rather than relying on a binary classification of normal versus tumor, we aim to provide more precise and useful guidance for complete excision during surgery. This approach not only aids in margin assessment, but could also assist the surgeon in identifying the specific type of tissue, enhancing surgical decision-making and future radiation or chemotherapy treatment strategy.

Raman spectroscopy offers several advantages as a diagnostic tool in tissue analysis and classification. It is non-invasive and provides detailed molecular information, enabling clinicians to identify specific biochemical changes in tissues. These capabilities make Raman spectroscopy highly valuable for distinguishing among malignant, intermediate and benign tumors. However, in-vivo Raman spectroscopy measurements (probe-based) face limitations from the small number of spectra that can be collected during a single session. This constraint can affect the comprehensiveness and accuracy of the analysis, as fewer data points may not fully capture the heterogeneity of the tissue being examined. In contrast, microscopic Raman spectroscopy enables the acquisition of thousands of spectra from a small area, providing high-resolution molecular information. This extensive dataset enhances the precision of tissue characterization and differentiation.

Accordingly, this study aims to investigate the potential of microscopic Raman spectroscopy for distinguishing among STS, benign tumors, and normal tissue types. Ex-vivo studies utilizing microscopic Raman spectroscopy can advance future deep learning algorithms by providing a rich spectral dataset. This comprehensive dataset can serve as a valuable reference for developing various deep learning applications, such as pre-processing, tissue-type classification, regression, and feature highlighting algorithms. The detailed spectral data obtained from ex-vivo studies can ultimately improve the reliability and effectiveness of Raman spectroscopy as a diagnostic tool in clinical settings.

## Materials and methods

### Sample preparation

All tissue samples were frozen and 10 *μ*m sections were cut using a Cryostat machine (Leica Biosystems, Deer Park, IL). Each section was placed on a stainless-steel plate for Raman analysis. Additional 5 *μ*m sections from the same tissue blocks were prepared for hematoxylin and eosin (H&E) staining and histologic assessment. These stained sections were used to identify the ground-truth label and target specific regions within the tissue samples for microscopic Raman measurements. Also, the pathologists in this study used these slides to confirm both the negative surgical margins and the specific tissue type.

### Data acquisition and conditioning

A Renishaw inVia confocal Raman microscope was used to acquire Raman spectra at a wavelength of 633nm He/Ne and an output power of 17mW. For each selected region, a 100 *μ*m × 100 *μ*m area was scanned using a 51 × 51 grid. Each exposure targeted a spot with a diameter of less than 1 *μ*m and an exposure time of 0.5 seconds, producing 2,601 Raman spectra for a rapid, single-spot-based tissue analysis in each selected area.

Data processing was performed using WIRE Software (Windows-based Raman Environment software, Renishaw Inc). Each Raman spectrum underwent baseline correction using a fifth-order polynomial, followed by median filtering to remove cosmic ray artifacts caused by high-energy particle-induced spikes. Noise smoothing was carried out using a Savitzky-Golay filter with a polynomial order of three, and each spectrum was then normalized to a range of 0 to 1. Finally, the spectra were truncated to the fingerprint region (wavenumber range of 400 cm^−1^ to 1800 cm^−1^) using Python, which contains features representing various vibrational modes of chemical bonds in organic molecules.

### Sample size

Following Stony Brook University Institutional Review Board approval, we recruited seven adult subjects scheduled for surgical excision from February 14, 2020 to May 5, 2021, and informed written consent was obtained from all participants. Five of the subjects were confirmed to have malignant tumors, while one was diagnosed with a benign tumor. In one subject, normal but no tumor tissue was collected due to the tumor’s small size. The average age of the subjects was 65 years, with 57% being female. Tumors were excised from various locations, including the forearm, thigh, arm, and knee.

The number of subjects, number of measurements and tissue types are summarized in Table 1. All tissue subtypes in Table 1 follow the WHO classification for STSs [1].

**Table 1:** Sample types and spectra counts for each tissue type.

Tissue Type	Abbreviation	Subtype	Subjects	Measurements
Muscle	MSC	Normal	4	33,712
Skin layers	SKN	Normal	5	62,424
Fat	FAT	Normal	3	31,212
Leiomyoma	LEM	Benign smooth muscle tumor	1	10,404
Myxoid liposarcoma	MLS	Malignant adipocytic tumor	1	83,742
High-grade pleomorphic liposarcoma	PLS	Malignant tumor of uncertain differentiation	2	28,764
Leiomyosarcoma	LEI	Malignant smooth muscle tumor	1	18,207
High-grade myxofibrosarcoma	HMS	Malignant fibroblastic/myofibroblastic tumor	1	18,207

### Deep learning analysis

A custom convolutional neural network (CNN) inspired by the Residual neural network (ResNet) architecture [21] was developed for the classification task. The ResNet design incorporates residual connections to address challenges such as vanishing gradient and accuracy degradation in deep networks [22,23]. The network begins with an initial convolutional layer followed by batch normalization, ReLU activation, and max pooling, which extract low-level features and reduce dimensionality. This is followed by four sequential layers, each containing two residual blocks.

**Fig 1:**
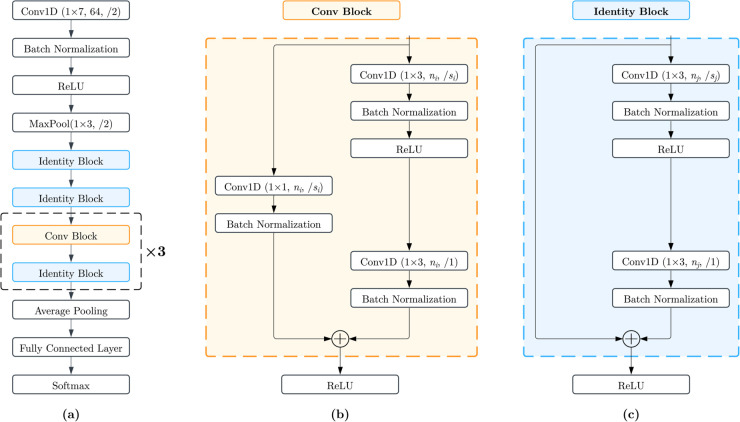
The proposed ResNet architecture. (a) illustrates the overall network structure, with Convolutional blocks shown in orange and Identity blocks shown in blue. (b) shows a Convolutional block, which includes an additional convolution and batch normalization layer in the shortcut path to match input and output dimensions. (c) depicts an Identity block, where the input bypasses the convolutional stack via a skip connection. Conv1D (1×*k*, *n*, /*s*) represents a 1D convolutional layer with kernel size *k*, number of filters *n*, and stride *s*. The network hyper parameters are *n*_*i*_ = {128,256,512}, *s*_*i*_ = {2,2,2} for Convolutional blocks, and *n*_*j*_ = {64,64,128,256,512}, *s*_*j*_ = {1,1,2,2,2} for Identity blocks.

The residual connections are implemented using two types of sub-blocks: the Identity block and the Convolutional block. Each block consists of a series of convolution, batch normalization, and activation layers, with the input bypassing the convolutional stack via a shortcut connection. The key difference between the two blocks is that the Convolutional block includes an additional convolution and batch normalization layer in its shortcut path. This ensures that the input and output dimensions match, allowing the skip connections to be added to the block’s output. The Convolutional block is used whenever hyper parameters such as filter size or strides need to be adjusted, as a network composed solely of Identity blocks cannot handle such changes without mismatched dimensions. Strides are implemented through the Convolutional Block to reduce computational cost and prevent overfitting. The network ends with an adaptive average pooling layer followed by a fully connected layer with softmax activation to output the classification probabilities for eight tissue types. The overall architecture of the proposed ResNet is shown in Fig 1.

The proposed network was implemented using Pytorch in Python and trained end-to-end using cross-entropy loss with class weights to address the dataset imbalance. The weights were inversely proportional to the class frequencies, ensuring that underrepresented tissue types contributed more significantly to the loss. A stochastic gradient descent (SGD) optimizer is used with a learning rate of 0.001 and a momentum of 0.9. To further enhance training efficiency, a cyclical learning rate (CLR) scheduler is employed [24]. The CLR scheduler varies the learning rate cyclically between a base learning rate of 0.001 and a maximum learning rate of 0.01, following a triangular policy. The step size for the scheduler is set to 8×1792, where 1792 is the number of training batches per epoch.

The dataset, consisting of 286,672 Raman spectra, was divided into 80% (229,330 spectra) for training and 20% (57,342 spectra) for testing, with an even distribution across each tissue type. The proposed ResNet model was trained for 50 epochs, and the model with the minimum validation loss was selected as the best-performing model. The training process was completed in approximately 30 minutes using an NVIDIA RTX A6000 GPU.

### Clinical alert metric

We introduced a clinical alert (CA) metric to enhance the assessment of our model’s performance in diagnosing malignant soft tissue sarcomas (STS). In addition to conventional evaluation metrics, such as sensitivity, specificity, and precision, clinical alert focuses on cases where a malignant STS is misclassified as normal or benign tissue. This metric is crucial, because such misclassifications can lead to significant clinical consequences, making it imperative to minimize this number. Failure to accurately diagnose a malignant STS could lead to delayed or improper treatment, potentially allowing tumor progression, metastasis, and a substantial decrease in patient survival rates. The clinical alert metric is calculated using the following equations:

$${\text{FN}}_{\text{C}}=\text{FN}-{\text{FN}}_{\text{M}}\;\text{CA}=\frac{{\text{FN}}_{\text{C}}}{\text{TP}+\text{FN}}$$ (1)

Here, ${\text{FN}}_{\text{C}}$ represents the clinical false negatives, calculated by subtracting the number of false negatives that were misclassified only as another malignant type (${\text{FN}}_{\text{M}}$) from the total false negatives (FN). This distinction is important because it focuses on the most critical errors, those where malignant cases are mistaken for non-malignant ones. The clinical alert ratio (CA) is then calculated by dividing the clinical false negatives by the sum of true positives (TP) and false negatives (FN), providing a clear measure of the model’s ability to correctly identify malignant cases. A low clinical alert (CA) score helps ensure that the model is not only accurate but also reliable in clinical settings, where the cost of misclassification can be high. However, in cases where the average clinical alert is particularly high, it is important to validate the model’s predictions by collecting multiple spectra from different locations, rather than relying on a single measurement. This redundancy enhances the reliability of the diagnostic process in clinical settings.

## Results

**Fig 2:**
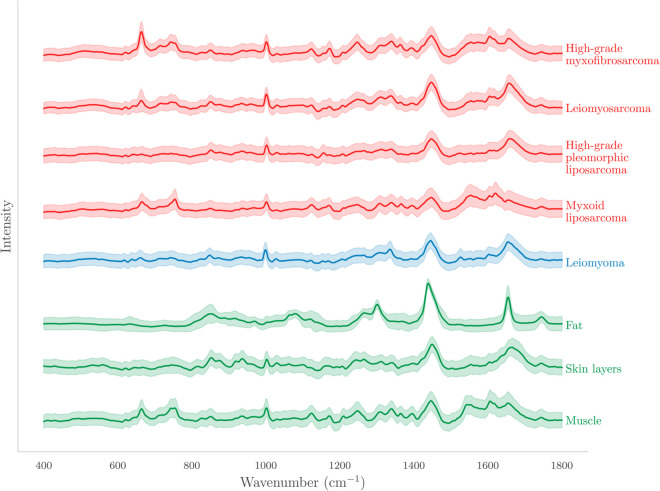
Averaged Raman spectra for all tissue types following pre-processing, with bands indicating one standard deviation, have been shifted vertically for clarity. The spectra are color-coded for clarity: green, blue and red represent normal tissues, benign tumors, and malignant tumors, respectively.

Following the data acquisition and pre-processing steps, we analyzed 286,672 Raman spectra from seven patients across eight tissue types. The averaged Raman spectra for each tissue type, along with their standard deviation intervals, are shown in Fig 2. Distinct spectral profiles were observed across tissue categories, with notable differences in peak intensities and positions. For example, normal fat tissue had prominent peaks at approximately 1300 cm^−1^, 1438 cm^−1^, and 1654 cm^−1^, while myxoid liposarcoma, a malignant adipocytic tumor, showed additional peaks near 1000 cm^−1^ and 1554 cm^−1^ that were absent in normal fat. High-grade myxofibrosarcoma, myxoid liposarcoma and leiomyosarcoma had distinct spectral signatures, particularly in the band range of 650–850 cm^−1^, contrasting with the spectral profiles of benign leiomyoma, fat, and skin layers. These differences in peak patterns enabled clear visual separation of malignant, benign, and normal tissues in the averaged spectra. The model achieved an overall accuracy of 97.1% with a weighted average precision, recall, and F1-score of 0.971 across all tissue types (Table 2). Sensitivity values ranged from 93.2% for high-grade myxofibrosarcoma to 100% for leiomyoma, while specificity ranged from 99.1% for myxoid liposarcoma to 100% for leiomyoma.

**Table 2:** Summary of sensitivity, specificity, precision, and clinical alert for each tissue type.

Tissue Type	Sensitivity (%)	Specificity (%)	Precision (%)	Clinical Alert (%)
Muscle	95.5	99.5	96.4	NA
Skin layers	95.7	99.2	97.2	NA
Fat	99.6	99.9	99.4	NA
Leiomyoma	100.0	100.0	100.0	NA
Myxoid liposarcoma	97.9	99.1	97.8	0.997
High-grade pleomorphic liposarcoma	98.0	99.5	95.6	1.981
Leiomyosarcoma	97.6	99.7	96.0	2.389
High-grade myxofibrosarcoma	93.2	99.5	92.6	1.812

The clinical alert (CA) metric, which quantifies the misclassification of malignant cases as benign or normal tissue, yielded an overall rate of 1.46%. Among malignant subtypes, leiomyosarcoma had the highest clinical alert rate (2.39%), while myxoid liposarcoma had the lowest (0.997%). These results underscore the model’s reliability in distinguishing malignant from non-malignant tissues, a critical factor for intraoperative decision-making.

**Fig 3:**
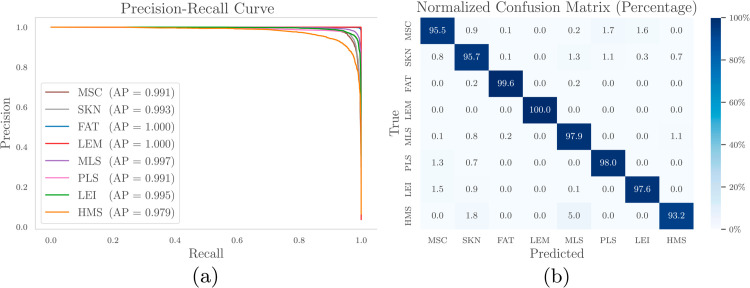
Classification results. (a) Precision-Recall curves (PRC) for all tissue types, showing area under the curve (AUC) or Average Precision (AP) values. (b) Confusion matrix of the classification model’s performance. Tissue type abbreviations: muscle (MSC), skin layers (SKN), fat (FAT), leiomyoma (LEM), myxoid liposarcoma (MLS), high-grade pleomorphic liposarcoma (PLS), leiomyosarcoma (LEI), and high-grade myxofibrosarcoma (HMS)

Precision-Recall curves (PRC) were generated to evaluate classification performance across tissue types (Fig 3a). The area under the curve (AUC) exceeded 0.98 for all categories, with leiomyoma achieving perfect discrimination (AUC = 1.00). In contrast, high-grade myxofibrosarcoma showed the lowest AUC (0.98). The confusion matrix (Fig 3b) revealed that 97.1% of spectra were correctly classified, with leiomyoma and normal fat achieving 100% and 99.6% sensitivity, respectively. High-grade myxofibrosarcoma was misclassified as myxoid liposarcoma in 5% of cases. In addition, 1.8% of high-grade myxofibrosarcoma spectra were confused with skin. Leiomyosarcoma that exhibited the highest clinical alert rate, primarily misclassified as normal muscle tissue. This could be due to the histologic origin of leiomyosarcoma as a malignant smooth muscle tumor, which may have biochemical similarities to normal muscle.

## Discussion

This study investigated the use of microscopic Raman spectroscopy to differentiate among soft tissue sarcomas, benign tumors, and normal tissue. Ex-vivo Raman measurements were performed on surgical samples from seven patients, producing 286,672 spectra using a 633 nm excitation wavelength. A custom CNN ResNet model achieved a 97.1% weighted accuracy in tissue classification, with a clinical alert rate of 1.46%. These results suggest that single Raman spectra could serve as a rapid, non-invasive tool for distinguishing between normal and abnormal tissues at surgical margins. The study also generated a comprehensive spectral dataset to support advanced deep learning model development.

Our results demonstrate the ability of Raman spectroscopy to differentiate among various tissue types. This capability stems from the unique Raman spectra, which serve as biological fingerprints of tissues, reflecting the distinct chemical bonds and molecular structures within them. For example, fat spectra show distinct peaks at 1266, 1300, 1438, and 1654 cm^−1^ due to the vibrational modes of the C-C, C-O, =C-H, CH2, CH3, and C=C groups in lipids [7,25,26]. In previous work, the ratio of bands at 1660/1445 cm^−1^ was used to determine the unsaturation of lipids [27,28]. Liposarcomas, malignant adipocytic tumors, share several prominent spectral peaks with normal fat tissue. However, they also have additional characteristic bands at about 1554 cm^−1^ and 1000 cm^−1^, which are attributed to carotenoid vibrational modes. As reported by Manoharan et al. a particularly notable spectral distinction between normal adipose tissue and liposarcoma lies in the intensity ratio of the bands at 1440 cm^−1^, associated with C-H bending, and 1657 cm^−1^, corresponding to C=C stretching. In the tumor spectrum, the increased intensity of the C=C band relative to the C-H band indicates a higher degree of lipid polyunsaturation. This increase in lipid polyunsaturation is observed as tissue transitions from normal to malignant states. In addition, the intensity ratio appears to correlate with the grade of the tumor [29]. The findings from our study align with a similar trend, demonstrating a higher increase in lipid polyunsaturation in pleomorphic and myxoid liposarcomas compared to normal adipocytic tissue.

As shown in Fig 2, normal muscle and several STS subtypes like high-grade myxofibrosarcoma, leiomyosarcoma, and myxoid liposarcoma, have a strong band between 650 and 850 cm^−1^. This band includes a range of peaks commonly associated with amino acids and nucleotides [7,30–32]. Overall, close similarities can be observed among many of the STS subtypes, particularly with major and minor peaks appearing at 1438 and 1654 cm^−1^. However, the classification CNN model demonstrated the ability to go beyond relying solely on the intensity/ratio of these two major peaks, indicating the presence of numerous other potential biochemical markers associated with STS. This suggests that the model captures a more complex and comprehensive biochemical signature, offering deeper insights into the molecular characteristics of STS.

A prior study on STS using near-infrared in vivo Raman spectroscopy reported 89.5% sensitivity and 96.4% specificity for differentiating STS from normal muscle and fat, excluding well-differentiated liposarcomas [7]. Our results extend these findings by incorporating deep learning, which improves classification accuracy and captures a more detailed biochemical signature. In addition, the extensive spectral dataset generated in our study serves as a valuable resource for future research. Large datasets are essential for training deep learning models, ensuring better generalization and performance. Unlike studies that relied on synthetic data due to limited sample availability, such as a GAN-based approach that improved skin cancer classification by generating synthetic Raman spectra [33], our comprehensive dataset eliminates the need for synthetic augmentation. This dataset not only ensures robust model generalization but also serves as a strong baseline for developing deep convolutional neural networks aimed at refining pre-processing techniques for Raman spectroscopy data [34]. By addressing prior limitations related to data scarcity and model reliability, our work contributes to advancing deep learning applications in Raman-based diagnostics.

In contrast to frozen section margin biopsy examination, the current standard method that typically takes 30–60 minutes to complete [8], Raman spectroscopy provides a rapid, non-destructive alternative for evaluating tumor margins and tissue types. Our model is specifically trained on individual Raman spectra, meaning that a single spectrum, acquired in just a few seconds, provides sufficient information for accurate tissue classification. In addition, all evaluation metrics reported in our manuscript are derived from testing the model using individual spectra. This capability makes Raman spectroscopy a valuable tool for detecting abnormalities in tumor margins and guiding decisions on further excision or additional treatments. By significantly reducing evaluation time, it can improve the management of soft tissue sarcomas, enhancing surgical efficiency, and patient outcomes.

The findings of this study should be considered alongside several important limitations. While the methodology demonstrated strong performance in detecting specific soft tissue sarcoma (STS) subtypes, the analysis was restricted to a limited range of tissue types and a relatively small number of subjects. Although the total number of spectra collected was substantial, a broader range of tissue types across a larger group of subjects is essential for more reliable generalization of the results. Additionally, conventional Raman spectroscopy systems are inherently bulky, making them challenging to integrate into clinical workflows, particularly in real-time surgical settings where portability and ease of use are essential. However, the spectral fingerprints identified in this study could serve as a foundation for the use of compact, handheld Raman devices that are better suited for clinical environments. Another limitation is the requirement for sample preparation, which complicates its use in intraoperative scenarios. While these factors currently hinder immediate clinical translation, the study provides a foundation for optimizing Raman-based techniques and deep learning analysis for future diagnostic and surgical applications.

In summary, our study provides strong evidence that Raman spectroscopy, when integrated with deep learning, can accurately distinguish STS from normal and benign tissues. By significantly reducing processing time relative to standard frozen-section analysis, this approach offers a promising pathway for improving surgical margin assessments and patient outcomes. The spectral dataset we have compiled can further serve as a foundation for future refinements in Raman-based diagnostics, helping to shape more effective and practical clinical applications.

## Conclusion

The findings of this study demonstrate that microscopic Raman spectroscopy can effectively differentiate soft tissue sarcoma (STS) types from surrounding normal tissue. This was achieved by developing a ResNet-based classification algorithm, which was tested on 286,672 Raman spectra from seven patients. The proposed algorithm successfully classified eight distinct tissue types, achieving an overall accuracy of 97.1%, highlighting its potential as a powerful tool for precise tissue characterization and diagnosis. In addition, the model achieved a clinical alert (CA) value of 1.46%, emphasizing its reliability in minimizing critical misclassifications of malignant cases as non-malignant, which is crucial for ensuring accurate diagnosis in clinical settings. A key direction for future research involves validating our results using a handheld probe for in-vivo studies. This approach will enable real-time, non-invasive analysis of STS, potentially improving the accuracy and efficiency of sarcoma diagnosis during surgical procedures. Furthermore, the available microscopic Raman data collected in this study can serve as a valuable reference for developing and refining future classification algorithms. To further enhance the robustness and clinical applicability of these findings, future research should integrate data from a larger group of subjects and include a wider range of soft tissue sarcoma types and subtypes.
